# Proposed strategies for easing COVID-19 lockdown measures in Africa

**DOI:** 10.11604/pamj.2020.36.179.24194

**Published:** 2020-07-13

**Authors:** David Lagoro Kitara, Eric Nzirakaindi Ikoona

**Affiliations:** 1 Harvard T.H. Chan School of Public health, Department of Global Health and Population, Boston, Massachusetts, United States of America,; 2 Gulu University, Faculty of Medicine, Department of surgery, Gulu, Uganda,; 3 African Field Epidemiology Network, Freetown, Sierra Leone

**Keywords:** SARS-CoV-2, COVID-19 pandemic, Africa, lockdowns, situational analysis, evidence

## Abstract

As SARS-CoV-2 rapidly spread across the globe, short-term modeling forecasts provided time-critical information for containment and mitigation strategies. Global projections had so far incorrectly predicted large numbers of COVID-19 cases in Africa and that its health systems would be overwhelmed. Significantly higher COVID-19-related mortality were expected in Africa mainly because of its poor socio-economic determinants that make it vulnerable to public health threats, including diseases of epidemic potential. Surprisingly as SARS-CoV-2 swept across the globe, causing tens of thousands of deaths and massive economic disruptions, Africa has so far been largely spared the impact that threw China, USA, and Europe into chaos. To date, 42 African countries imposed lockdowns on movements and activities. Experience from around the world suggests that such interventions effectively suppressed the spread of COVID-19. However, lockdown measures posed considerable economic costs that, in turn, threatened lives, put livelihoods at risk, exacerbated poverty and the deleterious effects on cultures, health and behaviours. Consequently, there has been great interest in lockdown exit strategies that preserve lives while protecting livelihoods. Nonetheless in the last few weeks, African countries have started easing restrictions imposed to curb the spread of SARS-CoV-2. WHO recommends lifting of lockdowns should depend on the ability to contain SARS-CoV-2 and protect the public once restrictions are lifted. Yet, the greatest challenge is the critical decision which must be made in this time of uncertainties. We propose simple strategies on how to ease lockdowns in Africa based on evidence, disease dynamics, situational analysis and ability of national governments to handle upsurges.

## Commentary

**Introduction:** as SARS-CoV-2, a virus that causes a disease, COVID-19 rapidly spread across the globe, short-term modeling forecasts provided time-critical information for containment and mitigation strategies [1]. Global projections had so far incorrectly predicted large numbers of COVID-19 cases in Africa and that its health systems would be overwhelmed due to Africa´s vulnerabilities [1,2]. Significantly higher COVID-19-related morbidity and mortality were expected in Africa mainly because of the continent's poor socio-economic determinants that make it vulnerable to public health threats, including diseases of epidemic potential [1-3]. Surprisingly, as SARS-CoV-2 swept across the globe, causing tens of thousands of deaths and massive economic disruptions, Africa has so far been largely spared the kind of impact that threw China, the United States of America, and Europe into chaos [2,4].

**Adverse consequences of lockdowns in African countries**

**Figure 1:**
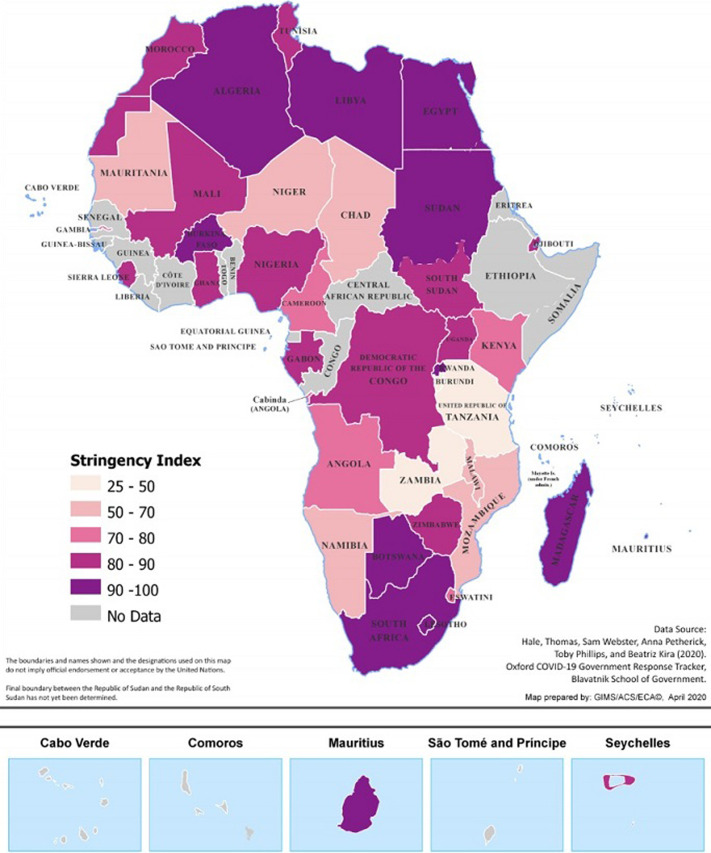
strictness of lockdowns in Africa as of 4 May 2020

**Economic and social effects:** to date, at least 42 African countries have imposed partial or complete lockdowns on movements and activities of population (Figure 1) [3,5]. Lockdown measures posed considerable economic costs that, in turn, threatened lives, put livelihoods at risk and exacerbated poverty [3]. A study conducted by the United Nation Economic Commission for Africa (ECA) established for example that lockdowns imposed extremely high costs on businesses and people, where up to 2.5% of the Gross Domestic Products (GDP) for Africa was estimated to be at risk every month [3]. African firms surveyed by ECA were reportedly being operated at only 43%, and that 70% of slum dwellers were missing meals or eating less as a result of COVID-19 pandemic [3]. Also, the imposition of lockdown measures to contain the exponential spread of COVID-19 adversely affected the unskilled and semi-skilled workers, international students, business persons, and patients on treatment abroad the most because, they were unprepared for a long stay outside their country including loss of livelihoods [3].

**Mental and health effects:** during the lockdown, disturbing images on the magnitude of violence, starvations, deprivations, mental health, depression, suicide and domestic violence that awash social and mainstream media in some Africa communities [3]. Major stressors that indubitably contributed to the widespread emotional distress and increased risks of psychiatric illnesses associated with COVID-19 have also been listed. This mainly resulted from uncertain prognoses, looming severe shortages of resources for testing and treatment, and for protecting responders and health care providers from infection, imposition of unfamiliar public health measures that infringed on personal freedoms (for example social and physical distancing and stay home policy), huge financial losses, and conflicting messages from authorities were among the many others [3]. These effects may have deciphered into wide ranges of emotional reactions (such as distress or psychiatric conditions), unhealthy behaviors (such as excessive alcohol and substance use), and non-conformity to public health directives (for example; home confinement, and physical distancing) in people who contracted the disease and in the general population [3]. Moreover, wide-ranging research on mental health in disaster has established that emotional distress is ubiquitous in affected population; a finding which is certain to be echoed in African populations affected by the COVID-19 pandemic [3]. Besides, there has been an observed reverse migration of some population from cities to rural areas since the COVID-19 outbreak and this is expected to have serious public-health implications (for example, increased community transmission of the disease and urban practices which are uncommon in rural areas e.g. prostitution) which should not be underestimated [3]. The lockdown measures have created throngs of problems in communities in many African countries and communities.

**Benefits of the lockdown measures in Africa:** experience from around the world suggests that lockdown interventions effectively suppressed the spread of COVID-19 in many African countries and that, lockdown measures forestalled severe vulnerabilities in African countries where there were only 1.8 hospital beds available per 1,000 people, and regionally, the potential risk of spread of SARS-CoV-2 was considered high because only 34% of the population in Africa had access to household facilities for hand washing with soap and water [3]. These lockdown measures strenuous as they were, difficult as they seemed and reported, provided the necessary protection to the African population at the most critical time of the COVID-19 pandemic. Consequently, there has been great interests in lockdown exit strategies, schemes which preserve lives but protect livelihoods. Many academicians, researchers, politicians and policy makers have expressed views on the challenges of making critical decisions in a situation of many uncertainties [3]. Accordingly, in the last few weeks, African countries for example; Nigeria, South Africa, Ghana, Kenya, Uganda and Senegal have started the process of easing the lockdown restrictions imposed to curb the spread of COVID-19 [3].

**Dilemma in exit strategy of lockdown measures in Africa:** of urgency are answers to questions on whether Africa could save its economy while at the same time protect its population from COVID-19 pandemic? The answers to these questions are not straightforward as different scholars have shown marked differences in options forward. In this paper, we propose evidence-based simple strategies that could help African governments rationally ease lockdown measures it imposed on citizens to protect the population from getting infected with SARS-CoV-2.

**Reasons and solutions for the strategies:** the proposed strategies attempt to answer questions on who, why, where, what, when and how to ease lockdown measures in Africa moving forward.

**Emphasis on basic epidemiological principles on prevention and control of an infectious disease:** our proposed strategies are premised on basic epidemiological principles of preventing infectious diseases from spreading into a population which include characterization of the epidemic by place, time, and person. It also includes getting answers to why those who get infected get infected by identifying risk factors, modes of transmission and vehicles of that infection [5]. Just like for any other infectious respiratory diseases, for SARS-CoV-2 it is key to identify cases, isolate them, and quarantine contacts for appropriate period to avert infecting the uninfected population. Besides, the population should adopt preventive measures such as regular hand washing with soap and water, using sanitizers, physical distancing, and wearing of face masks [5]. Congregate gatherings such as in; sports, markets, churches and mosques, funeral services, bars, public transport, weddings and schools should be limited as much as possible until the virus is contained [5,6].

**Enhanced shielding of the most at risk population:** current evidence has identified the elderly and persons with underlying medical conditions for example; diabetes, hypertension, heart diseases, obesity, cancer, renal diseases and other immunosuppressive conditions as the most vulnerable to SARS-CoV-2 [5,6]. There is need to protect the very high risk population in a strategy described as “enhanced shielding” which completely stops the most vulnerable population especially, the elderly and those with medical conditions stated above from getting into contacts with infected persons [6]. Also, it encourages that those visiting homes or sick bays of the elderly and those with severe medical conditions, are tested regularly to shield them from the most vulnerable population [6]. So, it is recommended that vulnerable people should be isolated from the general population by way of self-isolation, physical distancing and stay at home policy until the pandemic is put under control [6].

**Reproduction number (R0) for COVID-19:** one of the goals of the lockdown in Africa was to prevent the importation of new cases and in some countries to achieve a cut down of infection rate in order to force the reproduction number R0 (“R Naught”) to below 1 (a point at which the outbreak begins to decline) either by getting no more new cases of infection, recovery or dying. This was achieved by many African governments by instituting simultaneously, multiple public health interventions on the population [5].

**Classification of risks to COVID-19 transmission in a population:** differentiated lifting of restrictions in African countries depending on the level of risks to acquisition and transmission of SARS-CoV-2 to the population has been proposed [6]. This strategy of lifting restrictions could be based on low, moderate, and substantial risks of transmission of SARS-CoV-2 to the population [6]. Examples of low risk activities include outdoor physical exercises and jogging individually. Moderate risk places and activities include; opening up of shops, occasional gatherings of less than 10 people of a household in an outdoor setting, driving in personal cars, riding motorcycles (e.g. Boda-boda), farming, and herding cattle, and the like. On the other hand, substantial risks activities and places include; opening of public transport systems, working from outside homes, re-opening schools, bars, casinos, pubs, stadia, construction sites, places of worship such as churches and mosques, funeral processions, weddings, and huge parties such as street bashes and carnivores. This classification of activities and places according to risk of transmission is based on experience of lifting lockdowns in Wuhan Province in China which followed a gradual easing of restrictions by allowing residents to leave their residences on limited basis [6]. This was combined with massive screening program to test people at high risks of SARS-CoV-2 and everyone who had been in close contact with an infected person with the virus [6].

**Situational report on COVID-19 in Africa:** it is also evident that reporting only daily numbers of the positives, hospitalized cases and deaths provide limited insights into the state of COVID-19 pandemic in any African country or community. Many people may either develop no symptoms or have symptoms that are so mild that may not be detected through the health system-based surveillance which are being used in the African continent. The concentration of hospitalized cases in older individuals may lead to an idea that, there may be some widespread silent transmission among younger population. In the same way, where the majority of the population is infected and have recovered, it would mean the viral transmission would slow down thereby potentially reducing the need for stringent intervention measures currently in place in most African countries. Sadly, because we do not have adequate data from most African countries due to lack of enough tests, we are unable to draw any conclusion at this point.

**How to assess and decide on easing lockdowns in Africa:** how then do we assess and decide when to end the lockdown? Although the question on how to assess and decide on when the lockdown measures should end has become highly contentious among scholars, academicians, politicians, and policymakers, an African country planning to ease lockdown restrictions should have the ability to suppress the spread of SARS-CoV-2 sufficiently and that it would not produce a 5% surging wave of infection and deaths in the population in future. Also, an African country must be certain that it has passed the peak of the disease, and that the infection is declining, and it has not just temporarily suppressed the spread of the virus [1]. In mathematical modeling, this would mean the average number to which every infected person spreads the virus is able to fall or remain less than 1 [7]. Studies from around the world have calculated that the estimated value for reproduction number R0 (R Naught) for COVID-19 in a population is between 1.4 and 2.5 [7]. The R0 value is described as the transmission potential of an infectious disease and R0 < 1 indicates a low potential of extension capacity of an infectious disease. On the other hand R0 > 1 indicates a high potential of extension capacity of an infectious disease and therefore, there is need to institute control measures to limit its extension. Hence, bringing R0 < 1 may mean fewer people contracts SARS-CoV-2 than those recovering or dying from it, and so the number of new infections will decline and the epidemic may die out [7].

**Proposed strategies for easing lockdown measures in Africa based on gap analysis:** in our view, considering the simultaneous and multiple public health interventions already applied, and the deleterious effects of lockdowns on communities in Africa, and that strategies for coming out of lockdowns without hurting peoples´ health and livelihoods are not very well understood for an infectious disease that causes a pandemic, different scholars have postulated that safely opening up without adversely affecting the economy, health and livelihoods is a complex matter. For example, we need to find those who are infected through testing, case surveillance, contact tracing, isolation and quarantine as short and long-term measures. Also, Africa has many challenges with testing due to the limited test kits, underdeveloped laboratory services, expansive and diverse territories, inadequate human resources, poor road infrastructures, weak health systems, inadequate transport and poor referrals systems. Moreover, there is inadequate regional collaborations, investments in research, support from the population in financing and supporting responses to epidemics. Therefore, the following are the proposed strategies to African governments to consider as they ease lockdown measures to COVID-19 pandemic in their respective countries (Annex 1).

**Increase testing of all suspected cases, isolate and quarantine:** it is proposed that suspected cases are tested, isolated and quarantined in specific institutions or homes where practically possible until proven negative. Compared to all other continents, Africa has one of the least numbers of tests per capita conducted so far and so the number of COVID-19 cases presented now may not provide the actual number of COVID-19 cases in Africa. Increased testing of suspected cases, high risk groups, contacts, returnees from high risk countries and the general community members to detect sporadic cases to determine the actual number of cases in Africa is critical so as to allow policy makers decide whether to relax or re-apply the lockdown measures. This strategy requires a well-established community surveillance system, motivated health work force, with protective gears (personal protective equipment) (PPEs), adequate test kits, organized and coordinated systems of transfer of samples to the designated test sites (preferably using a hub system). It is also proposed that these test sites should be decentralized to regional centres where reports to and from test and source centres are timely delivered. Also, contact tracing of suspected and contact cases be conducted holistically and efficiently, avoiding spread of the virus but maintaining the human dignity by avoiding stigmatization of suspected and contact cases.

**Engage, sensitize and mobilize African population:** this strategy encourages national health authorities and countries´ leadership to dissemination preventive measures to the population through widely available means; televisions, radios, twitters, WhatsApp, Instagram, telegram and emails. This is essential because communities play important roles in epidemic control, their willingness to adhere to strenuous directives and compliance is critical. This has to be developed and coordinated in collaboration with COVID-19 National, provincial, district, parish and village task forces. Messages for engagement, sensitization and mobilization should be regular, factual and provided in a manner that does not cause anxiety, panic, depression and more uncertainties in the population. Also, this helps communities avoid stigmatization of those infected with COVID-19 because stigma creates undesirable effects to the response for example; it creates emotional, psychological, and mental harm. Besides, some of the infected persons may avoid seeking healthcare or may get discouraged from adopting healthy behaviours to the disease thereby creating more risks to the population [2].

**Regular communications, providing updates on the status of the country´s situation:** it is proposed that African countries should encourage their government agencies and Task forces to use mainstream and social media to inform and provide guidance on COVID-19 situational analysis at international, national and local levels respectively [2]. This is because communities need not be fed on fake news and inaccurate information because these hamper progress in the control of epidemics. Lessons learnt from the 40 year old experience with successful control of HIV should be one reference point where researchers and academicians recommended that the best way of handling the epidemic then, was by use of medical and nonmedical interventions including regular and factual behaviour change communication messages. Besides, the initial steps in the control of SARS-CoV-2 pandemic have been successfully undertaken by some African countries by proper and timely communication on the multiple public health intervention measures already listed above [3,5]. The current evidence suggests that SARS-CoV-2 transmission is perhaps greatest very early in the infection prior to the development of symptoms; the same lessons which were learnt from HIV at its early days when infected persons appeared normal (with no signs and symptoms of AIDS) as other community members. It is therefore proposed that since the routes of transmission of SARS-CoV-2 is known, biomedical and nonmedical prevention strategies that provide reliable protection become essential and should be timely communicated to the African population. Hence, behaviour change communications and regular updates on the virus becomes a crucial intervention that requires African governments to adopt.

**Create COVID-19 National, provincial, district, parish and village task forces:** these tiered taskforce system from the center to villages is proposed to support dissemination and follow-up of suspected cases in communities, providing an easy opportunity and effective mechanism for identification and follow-up of suspected cases. This is because most African countries have vulnerable and weak health care systems to sustain long-term and exhausting epidemic response. Task force members should be drawn from multi-stakeholder settings and should function to support health systems by picking expertise and experience from all stakeholders. It is further recommended that the flow of information from task forces are encouraged to be bi-directional, regular and coordinated by the line government ministry. Each task force should be supported by working subcommittees such as; Coordination and resource mobilization, case surveillance, research, risk communications, case management and infection prevention and control (IPC), logistics, security & enforcement, and water, sanitation and hygiene (WASH). It is expected that these task forces meet regularly perhaps once a week, updating members on progress in the prevention and control of the virus internationally, nationally and regionally, in addition to updating communities´ situations.

**Update the status of health facilities from National to rural centres:** it is imperative that health facilities plan, stock and pile the required tools, equipment and sundries for the management of COVID-19 cases. This is mainly because most African countries have health facilities that are poorly organized and facilitated to carry out their functions with persistent lack of essential materials for delivery of quality health care services. In addition, there is an observed increased in mental health problems in communities as a result of the lockdown. In easing the lockdown, African governments should scale up mental health services in all the health facilities including a community/grass root programs to mitigate the effects of this measure. More so, the purpose of this activity is to anticipate the likelihood, which may not be far-fetched that, there will be increased community transmission and severe cases of COVID-19 in African countries once the lockdown measures are lifted. In the case where some infected persons develop severe forms, institutions at the lowest level should be able to; identify, isolate and refer to regional centres efficiently. National, Regional, provincial and district health facilities must be well prepared, with the required resources to handle cases from lower level health centres. The idea is, once referrals are conducted timely ensuring no delays that cause panic, anxiety and uncertainties to COVID-19 cases, health workers and the public, it will restore confidence in the health systems in that community; an action which is key to epidemic control. In addition, a dedicated and well-motivated workforce in health facilities should be set aside to handle any COVID-19 cases preferably 24 hours a day until the outbreak is controlled.

**Procure and stockpile correct PPEs for all health workers:** this strategy is proposed to be adopted in each country, in all health facilities (Government and Non-government), and training of health workers on standard operating procedures (SoPs) for management of COVID-19. There are reports of severe shortages of PPEs and lack of training of health workers on the management of COVID-19 across the world but most especially in Africa. Many studies have shown the potential dangers of transmission of SARS-CoV-2 by infected health workers especially when they continue to treat non-COVID-19 patients [8]. It is estimated that an infected health worker increases vulnerability to the virus by creating more spheres of contacts and thus resulting into a full blown-out epidemic in that population. So, the availability of correct PPEs and ensuring that health workers use them to prevent infection are key to the pandemic control in Africa. “In addition, training of health workers on SoPs should be accompanied with a supervised use of PPEs every-time during work as studies have shown that nearly 50% of COVID-19 cases were asymptomatic [8]. Furthermore, Health workers in COVID-19 isolation units must be isolated from their families and housed, preferably in safe, secured housings designated for the purpose until the mandatory self-isolation period is completed [8].

**Strengthen active surveillance of COVID-19 cases:** it is key that active surveillance of SARS-CoV-2 particularly in densely populated areas, border points, schools, markets, and international truck drivers is regularly conducted. There is already evidence of active community transmission of COVID-19 in many African communities but not yet being reported due to lack of surveillance data. Active surveillance can be achieved by regular and mandatory testing of high risk groups to detect early asymptomatic cases using the village health teams or Community health extension workers (CHEWS) and providing the necessary remedies including isolation, quarantine and treatment as may be required [8]. In addition, African countries need to harness the flourishing mobile phone industry by innovating in mobile phone applications or web-based tools to provide self-guided tool for large population-level data collection which can help in tracing and follow-up of contacts and cases. This can be coxswained by partnering with major telecommunication companies that provide mobile phone network services and are widely distributed across the African Continent.

**Decentralize testing of SARS-CoV-2 to regional centres:** all efforts should be made to decentralize, ensure quality tests and assurance in regional centres. African countries are vast and sometimes with poor transport network which has been cited for delays experienced with returns of COVID-19 test results from a centralized test site due to long distances. This is premised on a goal that successful management of COVID-19 cases is better where testing and treatment of cases is conducted at the nearest points of case identification. The establishment of regional test and treat centres will reduce costs of transportation, undue delays and risk of spreading infection along the dreary referral processes. Also, this is likely to reduce congestion of cases (avoiding overflow and cross contaminations) in a single treatment center thus creating less spheres of risks of infection in the population.

**Allocate funds to stakeholders for competitive and feasible research on COVID-19:** an African country is likely to respond more suitably to the COVID-19 pandemic, if it understands SARS-CoV-2 spread, its dynamics, population characteristics and factors that determine spread [3,5]. This can best be determined by funding researches which are feasible and of high quality. Also, Institutions such as; Universities, Research organizations, health facilities, Nongovernmental Organizations (NGOs), bilateral and multilateral organizations must support research on all aspects including; health, economy, education, trade, behaviours and social life. Evidence adduced by researches provide the blueprints which feeds into the National task force, technical working groups and Ministry of health for policy formulation and implementation processes. It is key that African governments identify, earmark and fund studies on COVID-19 because there are so many uncertainties and unknowns about the virus and its effects on specific population groups. In addition, the virus has provided opportunity for review of vital synergies and challenges of sharing public health and scientific information, biological samples and genetic sequence data [9]. Also, it has been narrated that since the onset of the pandemic, thousands of SARS-CoV-2 sequences from around the world have been uploaded to the online databases such as the GenBank, and likewise, global initiative for sharing all influenza Data (GISAID) was done. The significance of data sharing on SARS-CoV-2 has helped in tracking spread, determined whether the containment strategies were successful and helped monitor the emergence of adoptive mutations in the viral genome [9]. These are commendable actions that promote scientific knowledge and shared actions which are very valuable in evidence-based decision making in epidemic control.

**Strengthen health referral systems:** one of the weakest links in health service delivery in many African countries is the referral links between the lowest health units and national referral centers. In order for African countries to overcome this challenge and develop systems which are sustainable, it is essential that it strengthens its referral systems to efficiently handle all COVID-19 cases and any emerging infectious diseases in the future. This necessitates health facilities to have adequate human resources, equipment, test kits, laboratory services and ambulances ready to transfer COVID-19 cases from one unit to the other. Laboratory services and hub systems should be buttressed to transfer biological materials while keeping the recommended standard operating procedures in place.

**Establish internal resource mobilization mechanisms:** for many years, African governments have relied on support and loans from major financial institutions such as the World Bank (WB) and International monetary fund (IMF) which are mostly conditional. With this pandemic and very difficult economic situation globally, and that most world economies are heading to recession, African governments should and must begin to plan and organize internal funding mechanisms using internally generated resources. Internal funding mechanisms must be put in place to support implementation of in-country COVID-19 strategic plan. Additionally, it is high time African governments focused on self-funding mechanisms as short and long-term measures in order to facilitate what they want to do and avoid conditionalities attached to international funding mechanisms. It is proposed that a special COVID-19 Trust Fund is set aside by each African governments in respective line government ministry to facilitate implementation of COVID-19 related programs for treatment, control and prevention.

**Create a COVID-19 National Control Program in the Ministry of Health:** to plan, execute, and handle health related issues to SARS-CoV-2. Several researchers, academicians, politicians and policy makers have predicted that SARS-CoV-2 will perhaps be around for many years to come unless eradicated by vaccination or other means. Also, several treatment protocols are at different levels of trials and there is no certainty on the timelines of approval. As for the availability of a vaccine, it is projected that, a safe, viable, affordable, accessible and mass vaccine for humans will come in months or even in many years to come [1,3]. That being the case, it is suggested that COPVID-19 National Control Program is established in the ministry of health and tasked to steer the program implementation in each country, designing policy frameworks for treatment, prevention and control of SARS-CoV-2 moving forward.

**Strengthen regional state collaboration on prevention and control of COVID-19:** policy approaches on how to handle COVID-19 pandemic have varied across nations and continents [5]. At the end of the day, humans travel and trade between and among each other in different countries. In many African countries for example, tribes and communities cut across borders and boundaries and they visit and share functions and activities together. So, collective approach by regional African governments would greatly help in the control, prevention and spread of COVID-19. For instance, East African countries approached the control of COVID-19 pandemic differently (complete lockdown versus no lockdown) and only to realize as a region that, one of the main drivers of transmission were the international truck drivers who were disproportionately affected with SARS-CoV-2 and, became the likely main drivers of the pandemic in East Africa [5]. So, for example, collaborative efforts among regional African countries would recommend testing of international truck drivers at the country of origin, border points and destinations. These collaborative actions would identify truck drivers with positive SARS-CoV-2 and take appropriate measures to control movements in the population to avoid spreading the virus [5].

**Institute and maintain night curfews and movement restrictions:** inspite of several warnings and alerts on the pandemic, a number of African population continued to participate in risky activities (heavy drinking in bars and night discos) as though there was no health problems of international concerns. So, in order to curtail these risky activities, some African countries imposed night curfews on residents which have been described by some scholars and commentaries in many parts of the world as controversial. However, considering the population behaviours in many African countries, restrictions by way of curfews appeared justified in some circumstances. This was to regulate and inform the public that it was not business as usual, the virus was still around and therefore utmost care had to be taken. In addition, after easing the current lockdown, continuing restrictions at risky places and activities are recommended to limit community spread of SARS-CoV-2, especially in hotspots with indications of community transmission. The duration of the curfew could however vary depending on COVID-19 situation analysis for each country or region. It is recommended that adjusted time for night curfews be put in place taking into consideration the incidence of COVID-19 and other urgent needs of the population. So, the decision on when and how to lift the curfews shall be left to the national authority to determine depending on the situations at hand.

**Strengthen global health security:** since the inception of global health security agenda (GHSA), US Centres for Disease Control and Prevention (CDC) have minimized public health threats by improving public health preparedness in USA and internationally. A number of countries are now investing in Global Health security, ensuring domestic preparedness, eliminating diseases and ending epidemics as their top priorities. It is commendable that CDC invested in 12 partner African countries to strengthen and sustain public health readiness to contain outbreaks at their sources. Accordingly, the areas that CDC focusses on are the four fundamental areas that underlie all aspects of global health security such as; surveillance system, laboratory systems, workforce development and emergency management and response. We argue that, with the development of African Centres for Disease Control and Prevention (ACDC), headquarter in Addis Ababa, Ethiopia, its high time African countries united to strengthen this center to partner and collaborate with the US CDC so that it can effectively scale up activities to cover the whole continent. In addition, similar replica of ACDC are created within member states with the view of strengthening the four pillars of Global Health Security in African Continent. Likewise, it was impressive to note that, ACDC have already accelerated its work to enhance diagnostic and surveillance capacity on the continent over the years [3]. Taking into consideration that epidemics know no borders, and success in controlling an epidemic in any one country will be limited if epidemics continue to rage elsewhere, it is key to support this global process by all countries in the African continent [3].

**Recommendation:** we recommend African governments to invest and focus on research, internal funding mechanisms and global health security. Epidemics are a great threat to humans and livelihoods.

**In conclusion** we propose simple strategies on how to ease lockdown measures in Africa based on evidence, disease dynamics, situational analysis and ability of national governments to handle upsurges. Investments in internal funding mechanisms, testing, research, health infrastructural development, mobile phone technology application utilization, multi-sectoral partnerships, health systems strengthening, community mobilization for health, regional collaborations and Global health security are strategic actions that will support African governments in short and long-term goals to overcome this COVID-19 pandemic and also prepare the African continent for any other disease with epidemic potential in the future.

## Annex

**Annex 1**: key ideas and features of the commentary paper
